# A meta-analysis of brain morphometric aberrations in adolescents who experienced childhood trauma

**DOI:** 10.3389/fnhum.2022.1022791

**Published:** 2022-12-06

**Authors:** Olga Tymofiyeva, Rebecca Hu, Roma Kidambi, Ca Nguyen, Jeffrey E. Max, Tony T. Yang

**Affiliations:** ^1^Department of Radiology and Biomedical Imaging, University of California, San Francisco, San Francisco, CA, United States; ^2^Department of Psychiatry and Behavioral Sciences, The Langley Porter Psychiatric Institute, University of California, San Francisco, San Francisco, CA, United States; ^3^Division of Child and Adolescent Psychiatry, Weill Institute for Neurosciences, University of California, San Francisco, San Francisco, CA, United States; ^4^Department of Psychiatry, University of California, San Diego, San Diego, CA, United States; ^5^Rady Children’s Hospital, San Diego, CA, United States

**Keywords:** childhood trauma, child maltreatment, adolescent brain, VBM, brain development

## Abstract

**Introduction:**

Childhood trauma is known to have dramatic effects on the risks for developing psychiatric disorders and increased suicidality. We conducted a meta-analysis of whole brain voxel-based morphometry (VBM) correlates of childhood trauma in adolescents exposed to childhood maltreatment (*N* = 379) and unexposed controls (*N* = 348).

**Methods:**

Anisotropic effect size-signed differential mapping (AES-SDM) was utilized to synthesize the studies.

**Results:**

We observed increased volume amongst adolescents with a history of childhood trauma in regions that are involved in motor functions and language production: left precentral gyrus, including part of the left inferior frontal gyrus, left fibers of the body of corpus callosum, and left postcentral gyrus. We observed decreased volume amongst adolescents with a history of childhood trauma in regions that are involved in language processing and/or sensory processing: bilateral cerebellum, bilateral middle temporal gyrus, left rostrum of corpus callosum, and bilateral supramarginal gyrus.

**Discussion:**

We suggest that these morphometric differences may be reflective of impaired motor development and increased sensory sensitivity and hypervigilance in adolescents with experiences of childhood trauma. Our results differ from meta-analytical findings in adults with history of childhood trauma and may contribute to a better understanding of neural mechanisms of childhood trauma, prediction of neurodevelopmental outcomes, and development of more effective and personalized therapies.

## Introduction

Trauma experienced during childhood is known to be strongly associated with risks for developing psychiatric disorders and increased suicidality (Angelakis et al., 2019; LeMoult et al., 2020; McKay et al., 2021). Understanding the neural mechanisms of childhood trauma may help explain the increased risk of suicidality, predict health outcomes, and offer better and more personalized treatments. Brain regions involved in emotion regulation are often implicated in both psychiatric disorders and childhood trauma. Specifically, meta-analyses of brain morphometry associated with childhood trauma indicate that aberrations in the prefrontal-limbic system, which encompasses the prefrontal cortex, hippocampus, and amygdala, could be a result of childhood trauma and an underlying cause for the subsequent development of mental disorders (Paquola et al., 2016).

Most of the morphometric studies of childhood trauma correlates, however, focused on adults. For a better understanding of a potential deviation from the normal neurodevelopmental trajectory after experiencing childhood trauma, neuroimaging studies need to be conducted earlier in life. Measuring childhood trauma correlates in adulthood may be confounded by the effects of aging and adult trauma exposure, as well as the accumulated effects that physical and mental illness can reciprocally have on brain structure. Mood disorders linked to childhood trauma such as Major Depressive Disorder (MDD) are especially likely to appear during adolescence (Di Martino et al., 2014). This warrants a special research focus on adolescent brains.

Indeed, adolescent brains differ from adult brains and may show different aberrations linked to childhood trauma compared to adults. For example, a meta-analysis by Paquola et al. (2016) reported that trauma cohorts (from 38 studies) exhibited smaller hippocampus and amygdala volumes bilaterally. The most robust findings of the whole brain voxel-based morphometry (VBM) meta-analysis were reduced gray matter in the right dorsolateral prefrontal cortex and right hippocampus amongst adults with a history of childhood trauma. Interestingly, meta-regression analysis showed that age did moderate results such that larger differences in amygdala gray matter were present in older samples (Paquola et al., 2016). The exclusion of child studies also altered the main findings in four other meta-analyses of gray matter correlates of childhood trauma (Woon and Hedges, 2008; Lim et al., 2014; Riem et al., 2015; Pollok et al., 2022). A recent meta-analysis by Pollok et al. (2022) uncovered gray matter volume effects associated with a wide range of early life adversities (including low socio-economic status, urban upbringing, maltreatment, prenatal selective serotonin reuptake inhibitors (SSRI) exposure, very low birth weight, family history of alcohol dependence, etc.) in the right hippocampus, right amygdala, and the left inferior frontal gyrus. In their sub-analysis of the combined young children and adolescent group (age range: 3.51–17.09 years) the findings were preserved in the right amygdala and hippocampus, but no result was found for adults (age range: 18.40–56.89 years) (Pollok et al., 2022).

It can be expected that findings in the adolescent brain would differ from those in young children, as well as adults. The expected differences can, as discussed above, be linked to a different neurodevelopmental stage and to potential confounding by the effects of aging and mental illness. An additional reason for expected differences is that adolescents may give a more accurate account of their childhood experiences (Newbury et al., 2018) than when they become adults (Widom and Shepard, 1996; Widom and Morris, 1997). Finally, knowledge of brain morphometric aberrations in adolescents who have experienced childhood trauma may be critical for development of new interventions, since adolescence is a second peak of neuroplasticity when language is well developed, and interventions can be especially effective (UNICEF Office of Research - Innocenti, 2017). All these factors warrant a special focus on adolescents in studying effects of childhood trauma.

Given the importance of the developmental aspect of childhood trauma effects, the goal of this study was to conduct a meta-analysis of brain morphometric aberrations in adolescents who have experienced childhood trauma.

## Materials and methods

### Literature search

The literature search was conducted through December 2021 in PubMed using the following search string within title/abstract: (“childhood maltreatment” OR “child abuse” OR “early stress” OR “childhood adversities” OR “childhood trauma”) AND (“structural gray matter” OR “voxel-based morphometry” OR “whole-brain” OR “whole brain” OR “voxel based morphometry” OR “structural grey matter” OR “gray matter volume” OR “grey matter volume.” Additional papers identified through citation searching were added to the screening step. Titles and abstracts were screened to determine whether articles met the following overarching inclusion criteria: original empirical research articles with measures of whole-brain gray matter and childhood trauma, conducted in a human adolescent sample (10 < age < 23). Additionally, the following exclusion criteria were used: fewer than 10 subjects, no control, no whole-brain VBM analysis, or peak coordinates of significant clusters in MNI or Talairach space were not reported and could not be obtained from the authors. Records were screened by two reviewers independently.

For the purposes of this review, we defined trauma as witnessing or experiencing emotional, physical or sexual abuse or emotional or physical neglect. We used an expanded definition of adolescence as an age range between 10 years (when puberty begins) and 23 years (Sawyer et al., 2018).

### Data extraction

From each included study, we extracted participants’ mean age, sex ratio, psychiatric diagnosis, and medications separately for subjects with and without exposure to childhood maltreatment/adversity, as well as maltreatment/adversity types, and peak voxel coordinates. Data were screened by two reviewers (authors RK and CN) independently. Any disagreement between the two reviewers was resolved by a third, independent reviewer (OT).

### Whole-brain analyses

The analysis was conducted using anisotropic effect size-signed differential mapping (AES-SDM version 5.142). This choice of methodology allowed us to compare our results to the results of the meta-analysis conducted in adult subjects by Paquola et al. (2016). AES-SDM combines peak coordinates and statistical parametric maps by using standard effect size and variance-based meta-analytic calculations (Radua et al., 2012, 2014). Random-effects models are used in which each study is weighted according to its sample size and variability. This method enables conjunction analyses to compare abnormalities between the study groups (subjects with childhood trauma compared to those without childhood trauma) based on the evaluation of effect sizes.

To conduct statistical inference, we used the recommended threshold of *p* = 0.005 with peak *z* > 1 and cluster extent of > 20 voxels (Radua et al., 2012). We used jackknife sensitivity analysis to assess the contribution of each study to the overall results and thus explore the robustness of the results. We considered results that lost significance in > 10% of iterations as non-robust. Publication bias was assessed using Egger’s test for asymmetry of the funnel plot for each significant peak voxel derived from the AES-SDM meta-analysis.

## Results

**FIGURE 1:**
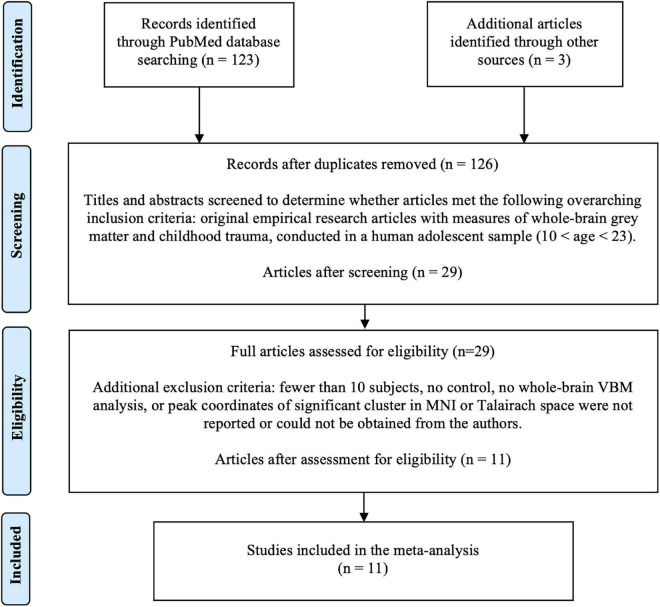
Literature search flow using PRISMA guidelines (Moher et al., 2009). VBM, voxel-based morphometry.

The initial literature search identified 123 studies (Figure 1). Three additional papers were identified through citation searching. After screening based on title and abstract review, 29 studies were selected for full text assessment for eligibility. After the assessment, 11 papers were included in the final analysis (Tomoda et al., 2009a,b, 2011, 2012; Liao et al., 2013; Lu et al., 2013; Walsh et al., 2014; Kelly et al., 2015; Fujisawa et al., 2018; Lim et al., 2018; Gao et al., 2021).

Summaries of sample characteristics of the included studies are described in Table 1. The total number of adolescents exposed to childhood maltreatment was 379. The total number of unexposed controls was 348.

**TABLE 1:** Summary of included articles.

	Exposed to childhood maltreatment (*N* = 379)						Unexposed control subjects (*N* = 348)				Main results
References	N	Mean age (years)	% Female	Psychiatric diagnosis	Maltreatment type	Maltreatment assessment	N	Mean age (years)	% Female	Psychiatric diagnosis	
Fujisawa et al. (2018)	21	12.8	61.9	RAD	Physical abuse, emotional abuse, sexual abuse, and neglect	TSCC	22	13.0	54.6	None	Reduction left primary visual cortex (BA 17)
Gao et al. (2021)	108	15.0	0	CD	Emotional abuse/neglect; physical abuse/neglect; sexual abuse	CTQ	74	15.1	0	CD	No change
Liao et al. (2013)	26	16.8	50.0	GAD	Emotional abuse/neglect; physical abuse/neglect; sexual abuse	CTQ-short form	25	16.8	50	GAD	No change
Tomoda et al. (2012)	22	21.8	72.7	Anxiety disorders, eating disorders, MDD, personality disorders, PTSD	Witnessed domestic violence	TAI	30	21.6	73.3	None	Reduction right lingual gyrus (BA 18)
Tomoda et al. (2009b)	23	21.7	34.8	ADHD (1 subject)	Corporal punishment	TAI, Life Experiences Questionnaire	22	21.7	72.7	None	Reduction right medial frontal gyrus (BA 10)
Tomoda et al. (2009a)	23	20.2	100	Depersonalization disorder, MDD, PTSD	Sexual abuse	TAI	14	19.0	100	None	Reduction bilateral primary and secondary visual cortex (BA 17–18)
Tomoda et al. (2011)	21	21.2	57.1	Anxiety disorders, MDD	Parental verbal aggression	TAI, Verbal Aggression Scale	19	21.1	63.2	None	Reduction left superior temporal gyrus (BA 22)
Walsh et al. (2014)	27	18.4	63.0	ADHD, CD, MDD, OCD, ODD	Emotional abuse, physical abuse, inter-parental discord	Cambridge Early Experiences Interview	31	18.4	48.4	None	Reduction cerebellum
Kelly et al. (2015)	62	12.2	46.8	None	Neglect, emotional abuse, physical abuse, sexual abuse	TSCC	60	12.7	58.3	None	Reduction bilateral medial temporal lobe, bilateral supramarginal gyrus, left medial orbitofrontal cortex. Increase left precentral gyrus
Lim et al. (2018)	22	17.6	31.8	ADHD, anxiety disorders, CD, depression, ODD, PTSD, social phobia	Selected for physical abuse (but severe emotional abuse and neglect also present)	CTQ	27/19	17.5/16.8	22.2	None/19 matched for psych. diag.	Reduction left lingual, pericalcarine, precuneus and superior parietal gyri; not dif. from psych. controls
Lu et al. (2013)	24	21.5	62.5	None	Emotional abuse/neglect; physical abuse/neglect; sexual abuse	CTQ	24	21.5	62.5	None	Reduction right middle cingulate gyrus

ADHD, attention deficit hyperactivity disorder; BA, brodmann area; CD, conduct disorder; CM, childhood maltreatment; CTQ, childhood trauma questionnaire; GAD, generalized anxiety disorder; MDD, major depressive disorder; OCD, obsessive compulsive disorder; ODD, oppositional defiant disorder; PTSD, post-traumatic stress disorder; RAD, reactive attachment disorder; TAI, traumatic antecedents interview; TD, typically developing; TSCC, trauma symptom checklist for children. All study subjects were unmedicated [only in the study by Kelly et al. (2015) medication status was not explicitly reported].

**FIGURE 2:**
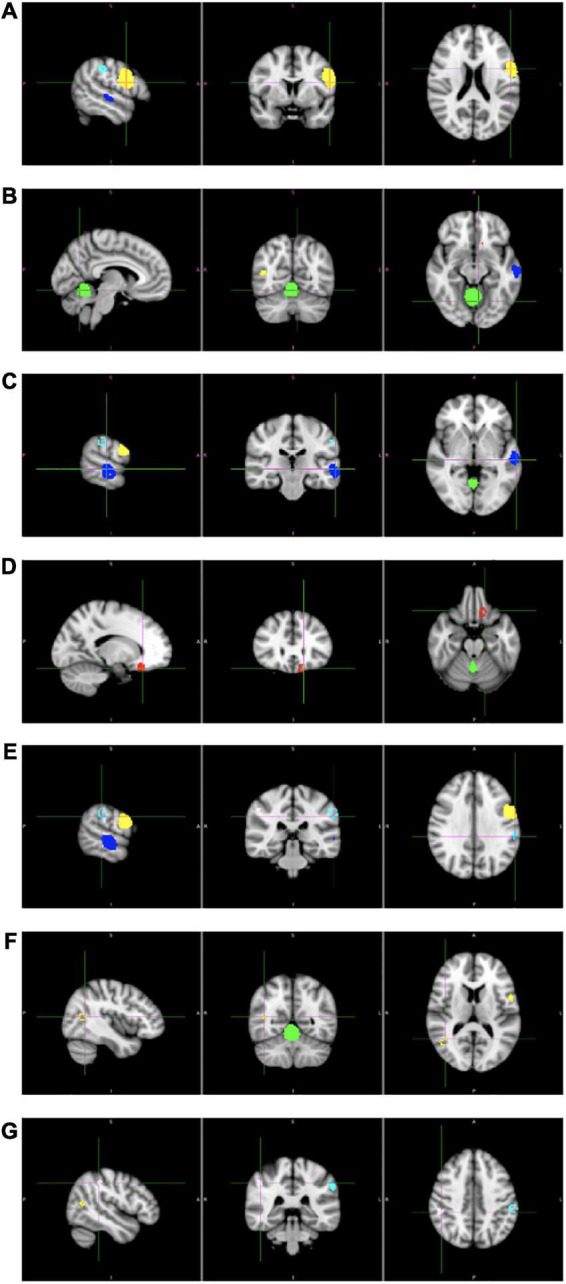
Results of whole brain voxel-based morphometry (VBM) meta-analysis of childhood trauma in adolescents. Clusters **(A–G)** corresponds to the clusters in Table 1.

The results of the meta-analysis are presented in Table 2 and in Figure 2. We obtained one cluster with significantly larger volume in adolescents with childhood trauma (Cluster A) and six clusters with significantly smaller volume in adolescents with childhood trauma (Clusters B–G). Cluster A included parts of the left precentral gyrus (including left inferior frontal gyrus, left fibers of the body of corpus callosum, left postcentral gyrus), and Clusters B–G included the bilateral cerebellum (including right lingual gyrus), bilateral middle temporal gyrus, left rostrum of corpus callosum, and bilateral supramarginal gyrus (including left inferior parietal gyrus) amongst adolescents with a history of childhood trauma. According to Jackknife analysis and Egger’s test, no single study was driving the reported endophenotype effect.

**TABLE 2:** Results of whole brain voxel-based morphometry (VBM) meta-analysis of childhood trauma in adolescents.

Peak MNI coordinate	Voxels	SDM *Z*-score	*P*	Hemisphere	Regions
**Clusters with larger volume in adolescents with childhood trauma compared to without trauma**					
−54, 4,20	1007	1.415	0.00003	L	Precentral gyrus, IFG, CC, postcentral gyrus, SLF, frontal aslant tract
**Clusters with smaller volume in adolescents with childhood trauma compared to without trauma**					
−6, −64, −12	1022	−1.783	0.00003	L, R	Cerebellum, lingual gyrus
−62, −24, −2	494	−1.696	< 0.00001	L	MTG, STG, CC, ARC
−16, 28, −22	149	−1.413	0.00116	L	CC, SFG, gyrus rectus, UNC, striatum, IFG
−60, −32,32	145	−1.463	0.00086	L	Supramarginal gyrus, IPG
42, −56,12	56	−1.408	0.00134	R	ARC, MTG, ILF
48, −36,42	39	−1.410	0.00127	R	Supramarginal gyrus, SLF

ARC, arcuate fasciculus; CC, corpus callosum; IFG, inferior frontal gyrus; ILF, inferior longitudinal fasciculus; IPG, inferior parietal gyri; L, left; MTG, middle temporal gyrus; R, right; SFG, superior frontal gyrus; SLF, superior longitudinal fasciculus; STG, superior temporal gyrus; UNC, uncinate fasciculus; VBM, voxel-based morphometry.

## Discussion

In the present study, we conducted a meta-analysis of brain morphometric correlates of childhood trauma in adolescents. Only studies with a whole-brain VBM analysis of differences between adolescents with and without history of childhood trauma were included, which resulted in 11 studies. The results indicate increased volume in the left precentral gyrus (including left inferior frontal gyrus, left fibers of the body of corpus callosum, left postcentral gyrus), and reduced volume in the bilateral cerebellum (including right lingual gyrus), bilateral middle temporal gyrus, left rostrum of corpus callosum, and bilateral supramarginal gyrus (including left inferior parietal gyrus) amongst adolescents with a history of childhood trauma.

Before offering any possible interpretation of the obtained results, we would like to discuss the methodological issue linked to VBM result interpretation in general. Decreased volume or thickness of gray matter obtained as a result of VBM is often interpreted as an actual thinning due to synaptic pruning and cell loss (Gogtay et al., 2004; Tamnes et al., 2010). However, cortical thickness estimates from MRI are based on the definition of the gray–white boundary. This boundary depends on the difference in T1 of white and gray matter, which is coupled with myelin content. Any misestimates of this boundary will lead to inaccuracies in estimating cortical thickness from MRI measurements. A recent study supports that, for example, the key source of apparent thinning of the human visual cortex during childhood is increased myelination of axons (Natu et al., 2019). By combining multiple quantitative neuroimaging methods and histology in postmortem data, Natu et al. (2019) provide evidence that the cortex does not thin during childhood but instead becomes more myelinated. Their results suggest that increased myelination during childhood changes the intensity of voxels on T1-weighted MRI and thus shifts the apparent gray–white matter boundary toward the cortical surface (Natu et al., 2019). This new evidence contradicts conclusions of previous research (Tamnes et al., 2010) but it has an improved methodology and validation.

In light of this understanding of VBM results in general, we would like to discuss the findings of our meta-analysis. All of our gray matter findings are located in brain regions that previously demonstrated apparent thinning in healthy youth aged 8–30 years (Tamnes et al., 2010). The finding of a thicker gray matter in youth with childhood trauma compared to those without childhood trauma may therefore reflect less myelination of axons adjacent to the regions, whereas thinner gray matter may reflect more myelination. We observed larger volume (potentially less myelination of axons adjacent to the regions) amongst adolescents with a history of childhood trauma in the left precentral gyrus (including left inferior frontal gyrus, left fibers of the body of corpus callosum, left postcentral gyrus). The precentral gyrus is associated with motor functions, whereas the opercular part of inferior frontal gyrus adjacent to the precentral gyrus in the left hemisphere is most likely associated with language production. Gvozdanovic et al. (2017) reported larger inferior frontal gyrus/precentral gyrus volumes were also related to an increased number of early intrusive film memories in healthy young females, as well as subjective distress and vividness of the intrusions. Changes in functional connectivity between the precentral gyrus and limbic regions have been reported in a trauma film group compared to a control film group during intrusive film picture presentation (Gvozdanovic et al., 2017). In another study, functional connectivity strength between bilateral precentral gyri and left amygdala positively correlated with the magnitude of reported physical abuse (Gvozdanovic et al., 2020). Finally, in an Affective Stroop task study, increased maltreatment (in particular abuse) was associated with decreased differential responsiveness of the precentral gyrus to incongruent task trials compared with view trials (Blair et al., 2019). On the behavioral level, the finding obtained in our study aligns with the recent call to consider physical developmental deficits in addition to cognitive, emotional, and social deficits when linking adverse childhood experiences to school-readiness (Wade et al., 2017). In a study by Wade et al. (2017), children with maltreatment showed rates of impaired motoric development five to seven times higher than expected, with those exposed to sexual or physical abuse having the highest rates. It has even been suggested that poor motor coordination may be causal in relation to emotional deficits such as anxiety, mediated by negative self-concept and low social support (Cairney et al., 2010). In addition to the primary motor cortex, the precentral gyrus also contains a portion of the supplementary motor cortex, which is involved in the planning of voluntary limb movement (Schott, 1993). A more speculative interpretation of the observed differences in our study is that they are related to tonic immobility—an involuntary motor and vocal inhibition reaction, considered the last-ditch response of the defensive cascade model, that can be induced by various types of traumatic events (Kalaf et al., 2017).

This meta-analysis also showed reduced volume amongst adolescents with a history of childhood trauma: in the bilateral cerebellum (including right lingual gyrus), bilateral middle temporal gyrus, left rostrum of corpus callosum, and bilateral supramarginal gyrus (including left inferior parietal gyrus). Almost all of these regions are involved in language processing and/or sensory processing. Since reduced apparent gray matter volume may be reflective of increased myelination of the adjacent white matter as described above, these results may be reflective of adaptive strategies in adolescents with experience of childhood trauma. In the case of abuse, children may become highly attuned to the environment and overly reliant on external cues (Mackiewicz Seghete et al., 2017). In the case of neglect sensory hypersensitivity is present less frequently than in the case of abuse and the opposite pattern of underresponsiveness is also observed (Howard et al., 2020), but an association with a heightened responsiveness to environmental stimuli, such as salient visual stimuli within visual cortices, has also been shown (Blair et al., 2019).

In summary, we suggest that the morphometric differences obtained in our study may be reflective of impaired motor development and increased sensory sensitivity and hypervigilance in adolescents who have experienced childhood trauma.

Interestingly, our results differ from the most robust findings of the whole brain VBM meta-analysis in adults with a history of childhood trauma by Paquola et al. (2016), who observed reduced gray matter in the right dorsolateral prefrontal cortex and right hippocampus. Their meta-regression analysis showed that age did moderate results such that larger differences in amygdala gray matter were present in older samples (Paquola et al., 2016). One possible interpretation is that, at first, the brain of a child experiencing trauma adapts to monitor threat, and over time the experienced childhood trauma takes a toll on other systems, creating more pronounced changes in the subcortical limbic structures and prefrontal cortex.

The interpretation of our results only partially aligns with a recent diffusion tensor imaging (DTI) meta-analysis that included child and adolescent samples along with adult samples and showed aberrations in structural connectivity associated with childhood trauma (Lim et al., 2020). Lim et al. (2020) found that maltreated individuals had significantly reduced fractional anisotropy (FA) in the left anterior thalamic radiation and bilateral fornix, optic radiations, inferior longitudinal fasciculus, and inferior frontal-occipital fasciculus, along with the anterior portions of the corpus callosum. There were no regions with increased FA. This lack of alignment can be due to the differences in demographics, age, and comorbidities. It can also be linked to the fact that white matter myelination close to the gray matter boundary might not be well-captured by FA, which gets closer to zero in the vicinity of the gray matter.

Our study should be interpreted in light of its limitations. The timing and the type of childhood trauma experienced (e.g., abuse vs. neglect) can be important (McLaughlin et al., 2019), as well as the accompanying psychiatric diagnosis (Table 1). Due to the limited sample size, we could not explore these variables —as well as other important variables such as sex and age—as moderators. In some of the included studies subjects who experienced childhood trauma had a psychiatric diagnosis, whereas the controls did not (Table 1). With small sample sizes and varied diagnoses within and across the included studies, it is difficult to disentangle brain differences related to childhood trauma and those related to specific psychiatric disorders. Interestingly, in the study by Lim et al. (2018), although there were no significant differences in the regions of interest (ROIs) between the abuse group and psychiatric controls or between the psychiatric and healthy controls, the brain measurements of the psychiatric controls were in between those of the abuse group and healthy controls. This suggests that the abuse group, by nature of the abuse experience *combined with* the psychiatric comorbidities, was more adversely affected than the psychiatric controls (Lim et al., 2018). The results of our meta-analysis also show a significant overlap with the results of a VBM-focused meta-analysis by Serra-Blasco et al. (2021), showing correlates of depression, anxiety, and post-traumatic stress disorder (PTSD) in regions such as left inferior frontal gyrus (L IFG), left superior temporal gyrus (L STG), superior frontal gyrus (SFG), fusiform gyrus [we found inferior longitudinal fasciculus (ILF) which connects to fusiform], and cerebellum, which all coincide with our findings. Although the meta-analysis by Serra-Blasco et al. (2021) was performed in a mixed population, the same group has published a protocol for a planned VBM-based meta-analysis of all major mental disorders and their comorbidities, in which they will have separate single linear models, one for children/adolescents and one for adults (Fortea et al., 2022). Our results combined with the knowledge that will be gained in this future meta-analysis could help shed light on the differences between manifestations of childhood trauma vs. specific psychiatric disorders in the adolescent brain.

An important strength of our study is its focus on adolescents. Previous meta-analysis excluded studies with individuals younger than 18 years (Paquola et al., 2016). Adolescent brains differ from adult brains and may show different aberrations linked to childhood trauma compared to adults. Moreover, adolescents may give a more accurate account of their childhood experiences than after they become adults. By restricting the age of studied subjects (age < 23 years) we addressed the potential confounding effects of aging and adult trauma exposure, and we addressed the accumulated effects physical and mental illness can have on brain structure.

The difference in findings between this meta-analysis in adolescents and prior meta-analytical findings in adults with a history of childhood trauma underlines the importance of examining adolescents and not assuming that adults and adolescents are the same. In mental health, data from adults are often applied to teens because of a critical lack of data in teens. Our results have important implications for the development of novel treatments for adolescents with psychiatric disorders (e.g., adolescent MDD), that could target brain regions associated with trauma and thus address a root cause potentially underlying psychiatric disorders and key transdiagnostic conditions such as suicidality. A better understanding of neural mechanisms of childhood trauma in adolescents may help develop such brain targets, offer more personalized therapies, monitor treatment effects, and predict neurodevelopmental outcomes.

## Data availability statement

The original contributions presented in this study are included in the article/supplementary material, further inquiries can be directed to the corresponding author.

## Ethics statement

Ethical review and approval was not required for the study on human participants in accordance with the local legislation and institutional requirements. Written informed consent from the patients/participants or patients/participants legal guardian/next of kin was not required to participate in this study in accordance with the national legislation and the institutional requirements.

## Author contributions

OT, RH, RK, CN, and TY conceived the work. OT, RH, RK, and CN conducted data collection. RH conducted data analyses. OT prepared the figures. OT and RH drafted the work. OT, RH, RK, CN, JM, and TY interpreted the data. All authors co-wrote the manuscript.

## References

[B1] Angelakis I. Gillespie E. L. Panagioti M. (2019). Childhood maltreatment and adult suicidality: a comprehensive systematic review with meta-analysis. *Psychol. Med.* 49 1057–1078. 10.1017/S0033291718003823 30608046 PMC6498789

[B2] Blair K. S. Aloi J. Crum K. Meffert H. White S. F. Taylor B. K. (2019). Association of different types of childhood maltreatment with emotional responding and response control among youths. *JAMA Netw. Open* 2:e194604. 10.1001/jamanetworkopen.2019.4604 31125109 PMC6632148

[B3] Cairney J. Veldhuizen S. Szatmari P. (2010). Motor coordination and emotional–behavioral problems in children. *Curr. Opin. Psychiatry* 23 324–329. 10.1097/YCO.0b013e32833aa0aa 20520549

[B4] Di Martino A. Fair D. A. Kelly C. Satterthwaite T. D. Castellanos F. X. Thomason M. E. (2014). Unraveling the miswired connectome: a developmental perspective. *Neuron* 83 1335–1353. 10.1016/j.neuron.2014.08.050 25233316 PMC4169187

[B5] Fortea L. Albajes-Eizagirre A. Yao Y.-W. Soler E. Verdolini N. Hauson A. O. (2022). Focusing on comorbidity—a novel meta-analytic approach and protocol to disentangle the specific neuroanatomy of co-occurring mental disorders. *Front. Psychiatry* 12:807839. 10.3389/fpsyt.2021.807839 35115973 PMC8805083

[B6] Fujisawa T. X. Shimada K. Takiguchi S. Mizushima S. Kosaka H. Teicher M. H. (2018). Type and timing of childhood maltreatment and reduced visual cortex volume in children and adolescents with reactive attachment disorder. *NeuroImage Clin.* 20 216–221. 10.1016/j.nicl.2018.07.018 30094171 PMC6080635

[B7] Gao Y. Jiang Y. Ming Q. Zhang J. Ma R. Wu Q. (2021). Neuroanatomical changes associated with conduct disorder in boys: influence of childhood maltreatment. *Eur. Child Adolesc. Psychiatry* 31 601–613. 10.1007/s00787-020-01697-z 33398650

[B8] Gogtay N. Giedd J. N. Lusk L. Hayashi K. M. Greenstein D. Vaituzis A. C. (2004). Dynamic mapping of human cortical development during childhood through early adulthood. *Proc. Natl. Acad. Sci. U.S.A.* 101 8174–8179. 10.1073/pnas.0402680101 15148381 PMC419576

[B9] Gvozdanovic G. Stämpfli P. Seifritz E. Rasch B. (2020). Structural brain differences predict early traumatic memory processing. *Psychophysiology* 57:e13354. 10.1111/psyp.13354 30825218

[B10] Gvozdanovic G. A. Stämpfli P. Seifritz E. Rasch B. (2017). Neural correlates of experimental trauma memory retrieval. *Hum. Brain Mapp.* 38 3592–3602. 10.1002/hbm.23613 28419641 PMC6866833

[B11] Howard A. R. H. Lynch A. K. Call C. D. Cross D. R. (2020). Sensory processing in children with a history of maltreatment: an occupational therapy perspective. *Vulnerable Child. Youth Stud.* 15 60–67. 10.1080/17450128.2019.1687963

[B12] Kalaf J. Coutinho E. S. F. Vilete L. M. P. Luz M. P. Berger W. Mendlowicz M. (2017). Sexual trauma is more strongly associated with tonic immobility than other types of trauma – A population based study. *J. Affect. Disord.* 215 71–76. 10.1016/j.jad.2017.03.009 28319694

[B13] Kelly P. A. Viding E. Puetz V. B. Palmer A. L. Mechelli A. Pingault J. B. (2015). Sex differences in socioemotional functioning, attentional bias, and gray matter volume in maltreated children: a multilevel investigation. *Dev. Psychopathol.* 27 1591–1609. 10.1017/S0954579415000966 26535946

[B14] LeMoult J. Humphreys K. L. Tracy A. Hoffmeister J. A. Ip E. Gotlib I. H. (2020). Meta-analysis: exposure to early life stress and risk for depression in childhood and adolescence. *J. Am. Acad. Child Adolesc. Psychiatry* 59 842–855. 10.1016/j.jaac.2019.10.011 31676392 PMC11826385

[B15] Liao M. Yang F. Zhang Y. He Z. Song M. Jiang T. (2013). Childhood maltreatment is associated with larger left thalamic gray matter volume in adolescents with generalized anxiety disorder. *PLoS One* 8:e71898. 10.1371/journal.pone.0071898 23951265 PMC3741188

[B16] Lim L. Hart H. Mehta M. Worker A. Simmons A. Mirza K. (2018). Grey matter volume and thickness abnormalities in young people with a history of childhood abuse. *Psychol. Med.* 48 1034–1046. 10.1017/S0033291717002392 29122037

[B17] Lim L. Howells H. Radua J. Rubia K. (2020). Aberrant structural connectivity in childhood maltreatment: a meta-analysis. *Neurosci. Biobehav. Rev.* 116 406–414. 10.1016/j.neubiorev.2020.07.004 32659288

[B18] Lim L. Radua J. Rubia K. (2014). Gray matter abnormalities in childhood maltreatment: a voxel-wise meta-analysis. *Am. J. Psychiatry* 171 854–863. 10.1176/appi.ajp.2014.13101427 24781447

[B19] Lu S. Gao W. Wei Z. Wu W. Liao M. Ding Y. (2013). Reduced cingulate gyrus volume associated with enhanced cortisol awakening response in young healthy adults reporting childhood trauma. *PLoS One* 8:e69350. 10.1371/journal.pone.0069350 23894454 PMC3722240

[B20] Mackiewicz Seghete K. L. Kaiser R. H. DePrince A. P. Banich M. T. (2017). General and emotion-specific alterations to cognitive control in women with a history of childhood abuse. *NeuroImage Clin.* 16 151–164. 10.1016/j.nicl.2017.06.030 28794976 PMC5540826

[B21] McKay M. T. Cannon M. Chambers D. Conroy R. M. Coughlan H. Dodd P. (2021). Childhood trauma and adult mental disorder: a systematic review and meta-analysis of longitudinal cohort studies. *Acta Psychiatr. Scand.* 143 189–205. 10.1111/acps.13268 33315268

[B22] McLaughlin K. A. Weissman D. Bitrán D. (2019). Childhood adversity and neural development: a systematic review. *Annu. Rev. Dev. Psychol.* 1 277–312. 10.1146/annurev-devpsych-121318-084950 32455344 PMC7243625

[B23] Moher D. Liberati A. Tetzlaff J. Altman D. G. (2009). Preferred reporting items for systematic reviews and meta-analyses: The PRISMA statement. *PLoS Med*. 6 10.1371/journal.pmed.1000097 21603045 PMC3090117

[B24] Natu V. S. Gomez J. Barnett M. Jeska B. Kirilina E. Jaeger C. (2019). Apparent thinning of human visual cortex during childhood is associated with myelination. *Proc. Natl. Acad. Sci. U.S.A.* 116 20750–20759. 10.1073/pnas.1904931116 31548375 PMC6789966

[B25] Newbury J. B. Arseneault L. Moffitt T. E. Caspi A. Danese A. Baldwin J. R. (2018). Measuring childhood maltreatment to predict early-adult psychopathology: comparison of prospective informant-reports and retrospective self-reports. *J. Psychiatr. Res.* 96 57–64. 10.1016/j.jpsychires.2017.09.020 28965006 PMC5725307

[B26] Paquola C. Bennett M. R. Lagopoulos J. (2016). Understanding heterogeneity in grey matter research of adults with childhood maltreatment-A meta-analysis and review. *Neurosci. Biobehav. Rev.* 69 299–312. 10.1016/j.neubiorev.2016.08.011 27531235

[B27] Pollok T. M. Kaiser A. Kraaijenvanger E. J. Monninger M. Brandeis D. Banaschewski T. (2022). Neurostructural traces of early life adversities: a meta-analysis exploring age- and adversity-specific effects. *Neurosci. Biobehav. Rev.* 135:104589. 10.1016/j.neubiorev.2022.104589 35189164 PMC9013474

[B28] Radua J. Mataix-Cols D. Phillips M. L. El-Hage W. Kronhaus D. M. Cardoner N. (2012). A new meta-analytic method for neuroimaging studies that combines reported peak coordinates and statistical parametric maps. *Eur. Psychiatry J. Assoc. Eur. Psychiatr.* 27 605–611. 10.1016/j.eurpsy.2011.04.001 21658917

[B29] Radua J. Rubia K. Canales-Rodríguez E. J. Pomarol-Clotet E. Fusar-Poli P. Mataix-Cols D. (2014). Anisotropic kernels for coordinate-based meta-analyses of neuroimaging studies. *Front. Psychiatry* 5:13. 10.3389/fpsyt.2014.00013 24575054 PMC3919071

[B30] Riem M. M. E. Alink L. R. A. Out D. Ijzendoorn M. H. V. Bakermans-Kranenburg M. J. (2015). Beating the brain about abuse: empirical and meta-analytic studies of the association between maltreatment and hippocampal volume across childhood and adolescence. *Dev. Psychopathol.* 27 507–520. 10.1017/S0954579415000127 25997768

[B31] Sawyer S. M. Azzopardi P. S. Wickremarathne D. Patton G. C. (2018). The age of adolescence. *Lancet Child Adolesc. Health* 2 223–228. 10.1016/S2352-4642(18)30022-1 30169257

[B32] Schott G. D. (1993). Penfield’s homunculus: a note on cerebral cartography. *J. Neurol. Neurosurg. Psychiatry* 56 329–333. 10.1136/jnnp.56.4.329 8482950 PMC1014945

[B33] Serra-Blasco M. Radua J. Soriano-Mas C. Gómez-Benlloch A. Porta-Casteràs D. Carulla-Roig M. (2021). Structural brain correlates in major depression, anxiety disorders and post-traumatic stress disorder: a voxel-based morphometry meta-analysis. *Neurosci. Biobehav. Rev.* 129 269–281. 10.1016/j.neubiorev.2021.07.002 34256069

[B34] Tamnes C. K. Ostby Y. Fjell A. M. Westlye L. T. Due-Tønnessen P. Walhovd K. B. (2010). Brain maturation in adolescence and young adulthood: regional age-related changes in cortical thickness and white matter volume and microstructure. *Cereb. Cortex N.Y.* 1991 534–548. 10.1093/cercor/bhp118 19520764

[B35] Tomoda A. Navalta C. P. Polcari A. Sadato N. Teicher M. H. (2009a). Childhood sexual abuse is associated with reduced gray matter volume in visual cortex of young women. *Biol. Psychiatry* 66 642–648. 10.1016/j.biopsych.2009.04.021 19560122 PMC4277202

[B36] Tomoda A. Suzuki H. Rabi K. Sheu Y. S. Polcari A. Teicher M. H. (2009b). Reduced prefrontal cortical gray matter volume in young adults exposed to harsh corporal punishment. *NeuroImage* 47(Suppl. 2), T66–T71. 10.1016/j.neuroimage.2009.03.005 19285558 PMC2896871

[B37] Tomoda A. Polcari A. Anderson C. M. Teicher M. H. (2012). Reduced visual cortex gray matter volume and thickness in young adults who witnessed domestic violence during childhood. *PLoS One* 7:e52528. 10.1371/journal.pone.0052528 23300699 PMC3530484

[B38] Tomoda A. Sheu Y. S. Rabi K. Suzuki H. Navalta C. P. Polcari A. (2011). Exposure to parental verbal abuse is associated with increased gray matter volume in superior temporal gyrus. *NeuroImage* 54(Suppl. 1), S280–S286. 10.1016/j.neuroimage.2010.05.027 20483374 PMC2950228

[B39] UNICEF Office of Research - Innocenti (2017). *The Adolescent Brain: A Second Window of Opportunity.* Florence: UNICEF Office of Research - Innocenti.

[B40] Wade T. J. Bowden J. Jane Sites H. (2017). Child maltreatment and motor coordination deficits among preschool children. *J. Child Adolesc. Trauma* 11 159–162. 10.1007/s40653-017-0186-4 32318146 PMC7163883

[B41] Walsh N. D. Dalgleish T. Lombardo M. V. Dunn V. J. Van Harmelen A. L. Ban M. (2014). General and specific effects of early-life psychosocial adversities on adolescent grey matter volume. *NeuroImage Clin.* 4 308–318. 10.1016/j.nicl.2014.01.001 25061568 PMC4107373

[B42] Widom C. S. Morris S. (1997). Accuracy of adult recollections of childhood victimization, Part 2: childhood sexual abuse. *Psychol. Assess.* 9 34–46. 10.1037/1040-3590.9.1.34

[B43] Widom C. S. Shepard R. L. (1996). Accuracy of adult recollections of childhood victimization: part 1. Childhood physical abuse. *Psychol. Assess.* 8 412–421. 10.1037/1040-3590.8.4.412

[B44] Woon F. L. Hedges D. W. (2008). Hippocampal and amygdala volumes in children and adults with childhood maltreatment-related posttraumatic stress disorder: a meta-analysis. *Hippocampus* 18 729–736. 10.1002/hipo.20437 18446827

